# Model-based estimation of the leaf area of ozone-indicator tobacco (*Nicotiana tabacum* L.) plants under ambient ozone conditions

**DOI:** 10.1016/j.mex.2023.102214

**Published:** 2023-05-10

**Authors:** Evgenios Agathokleous, Kun Zhang, Costas J. Saitanis

**Affiliations:** a School of Applied Meteorology, Nanjing University of Information Science and Technology (NUIST), Nanjing, Jiangsu 210044, China; b Lab of Ecology and Environmental Science, Agricultural University of Athens, Iera Odos 75, Athens 11855, Greece

**Keywords:** Ozone biomonitoring, Bioindicator plant, Leaf size, Linear regression, Predictive model, Bel-W3 leaf area calculation

## Abstract

Bel-W3 is an ozone-sensitive tobacco (*Nicotiana tabacum* L.) cultivar widely used worldwide for ozone biomonitoring. Despite its extensive use, there is no comprehensive predictive model to non-destructively estimate the leaf area using only a common ruler, yet leaf area is a major evaluative trait in plants under ozone stress and of economic value in tobacco plants. In this method, we aimed at developing a predictive model to estimate leaf area using the product between leaf length and leaf width. To this end, we conducted a field experiment with ground-grown Bel-W3 plants treated with different solutions under ambient ozone conditions. The solutions were water, the antiozonant ethylenediurea (EDU; 500 ppm), and the antitranspirant pinolene (Vapor Gard; 1%, 5%, 10%). The chemical treatments were introduced to enhance leaves pool and capture different conditions that can occur in ozone biomonitoring projects.

• A simple linear predictive model was developed and validated using data from a previous chamber experiment with small seedlings.
• Overestimation of the model led to the integration of data from both experiments and development of another simple linear predictive model.
• This integrated model provides improved estimation of leaf area and can be used for representative estimation of the area of Bel-W3 leaves of any sizes.

## Graphical abstract

**Figure:**
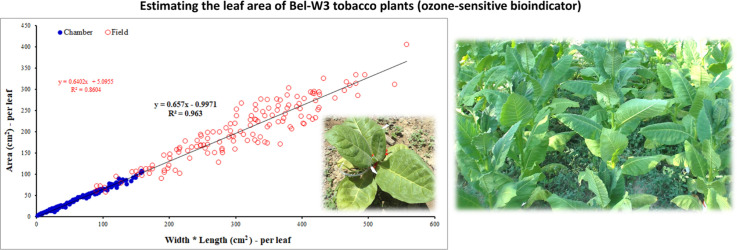

Specifications table

**Table:** 

Subject area:	Environmental Science
More specific subject area:	*Ozone biomonitoring*
Name of your method:	*Bel-W3 leaf area calculation*
Name and reference of original method:	*n/a (this is an original method)*
Resource availability:	*The data are presented in figures within the article*

## Method details

### Background

Ozone (O_3_) pollution is driven by anthropogenic emissions of ozone precursor gases under sunlight as well as weather conditions and presents a major environmental problem [1], [2], [3]. Ozone pollution is often enhanced during the summertime when ozone formation is favored by ideal weather conditions and, under specific urban situations, increased emissions of volatile organic compounds (VOCs) by vegetation along with decreased nitrogen oxides (NOx) emissions [4], [5], [6], [7]. Ozone high exposures commonly occur during the growing season of many types of plants, presenting a threat to vegetation due to the property of ozone to be a strong oxidant causing damage to living organisms [8], [9], [10], [11]. High ozone exposures negatively affect plant physiology (e.g. inhibiting photosynthesis) and growth, suppress yields of agricultural crops as well as productivity of (semi)natural vegetation, and disrupt fundamental ecological processes and services such as biodiversity, pollination, and nutrient cycling [12], [13], [14], [15], [16], [17], [18]. Therefore, it is important to monitor ozone effects on sensitive vegetation and protect plants against ozone damage [19], [20], [21].

Biomonitoring is an important technique that is used to record qualitative and quantitative changes in plants under specific environmental conditions in order to monitor ozone [22]. Besides its scientific and environmental value, biomonitoring can be used for promoting environmental education of the public and helping the development of regulation by providing important data [22]. Monitoring of ozone concentrations is often challenging, particularly in undeveloped countries or remote areas where electricity and/or the required financial support are unavailable, and biomonitoring with plants can be used to overcome these limitations [22], [23], [24]. In fact, ozone biomonitoring has been conducted extensively throughout the world in the last decades, mainly using ozone-sensitive and resistant genotypes of tobacco (*Nicotiana tabacum* L.), French bean (*Phaseolus vulgaris* L.), and white clover (*Trifolium repens* L.) [22,25]. The tobacco system is the oldest and the most extensively used, with successful results, among the ozone plant biomonitoring systems [22,25]. The tobacco system was developed by the Agricultural Research Service (ARS) at the USDA Agricultural Research Center (Beltsville, MD, USA) in the 1960s [23,26]. The ozone-sensitive cultivar commonly used within the tobacco biomonitoring system is Bel-W3 [23,26].

Along with foliar visible injury, growth and biomass are widely used as main indicators of ozone phytotoxicity. The leaf size is an important indicator of plant health, as ozone phytotoxicities can lead to microphyllia [from the Greek words «μικρός» (small) and «φύλλο» (leaf)], i.e. a condition in which leaves are smaller than they would normally be in the absence of ozone stress. On sensitive plants, such as of Bel-W3, extensive foliar visible injuries, such as discoloration and necrosis, are expected to result in adverse oxidative stress and thus growth inhibition. Moreover, the individual leaf area can be used to calculate the total plant leaf area, which is an integrated indicator of plant potential to harvest light (photosynthesize) and produce biomass and yield. In addition, individual leaf area is used to calculate the leaf mass per leaf surface area (LMA) or its inverse, specific leaf area (SLA), which is a functional trait determining plant sensitivity to ozone. Conversely to foliar visible injury, which is arbitrarily scored, leaf size is measured on a continuous scale and has absolute and accurate values. Therefore, combined assessment of foliar visible injury (as an ozone-exclusive sign) and individual leaf area (and thereby total plant leaf area) can provide insightful information about ozone exposures, phytotoxicities, and risk to vegetation. Furthermore, the plant leaf area in plants like tobacco is the harvestable product of commercial value, and thus calculating the actual plant leaf area degraded by ozone can reflect the actual yield loss, which can also be utilized to estimate the economic impact due to ozone phytotoxicity. However, there is no comprehensive predictive model for estimating leaf area of Bel-W3 plants non-destructively. Hence, this study aimed to develop an improved predictive model for estimating the leaf area of Bel-W3 ozone-bioindicator plants using data from simple non-destructive measurements of plant size (length and width).

## Experiment

To achieve the goal of research, an experiment was conducted at the experimental field of the Laboratory of Ecology and Environmental Science, at the campus of Agricultural University of Athens, in the metropolitan area of Athens, Greece (37°59′7.60″N; 23°42′23.32″E). Seeds of Bel-W3 were seeded on 25 February and kept in room temperature in the laboratory. The pot contained a commercially available soil substrate (Florabella, Klasmann–Deilmann GmbH, Germany) and was irrigated as needed to keep wet. Four days after seeding, the pot was transferred in a closed chamber with 14 L/10D hours photoperiod. On 24 March, each of 350 seedlings was transferred in a 500-cc pot filled with the same soil substrate. On 27 March, 120 uniform seedlings were placed at a semi-open penthouse for steady acclimation to outdoor conditions.

**Fig. 1:**
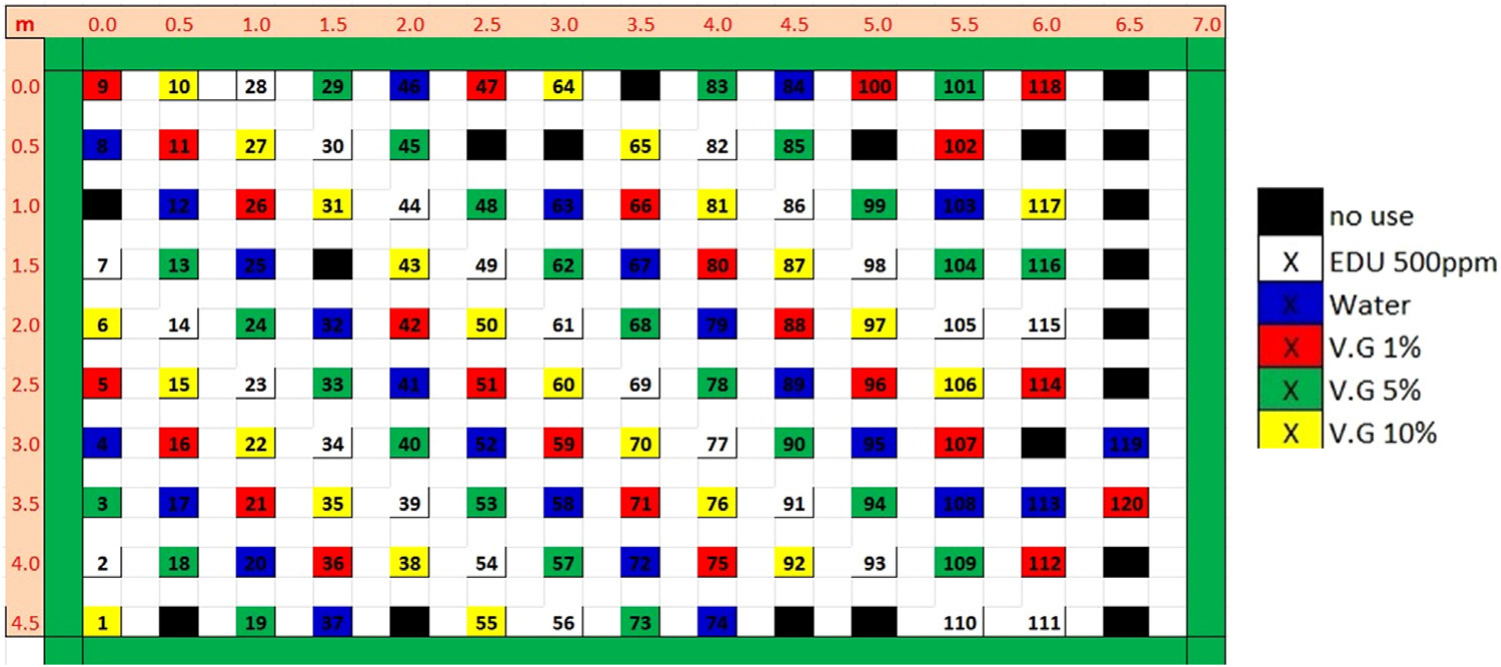
Experimental design. The distribution of Bel-W3 tobacco plants in the experimental field. The distance is indicated in meters (m). The integer numbers indicate the unique number of each of the 120 experimental plants. The plants were treated with water, ethylenediurea (EDU; 500 ppm), or (Vapor Gard (V.G.), a commercial di-1-*p*-menthene antitranspirant.

On 30 March, the plants were moved to the field. From this day, the plants were moved under shadow during the mid. day on a daily basis until transplantation to the experimental plot. On 2 May, foliar visible injuries from ozone were well noticeable on the majority of the plants, following an increase of average hourly concentrations of ozone to 60–80 ppb for some days (see succeeding text). On 3 May, transplantation of the plants from the pots to the ground of the experimental plot was carried out, and the experimental design is illustrated in Fig. 1.

**Fig. 2:**
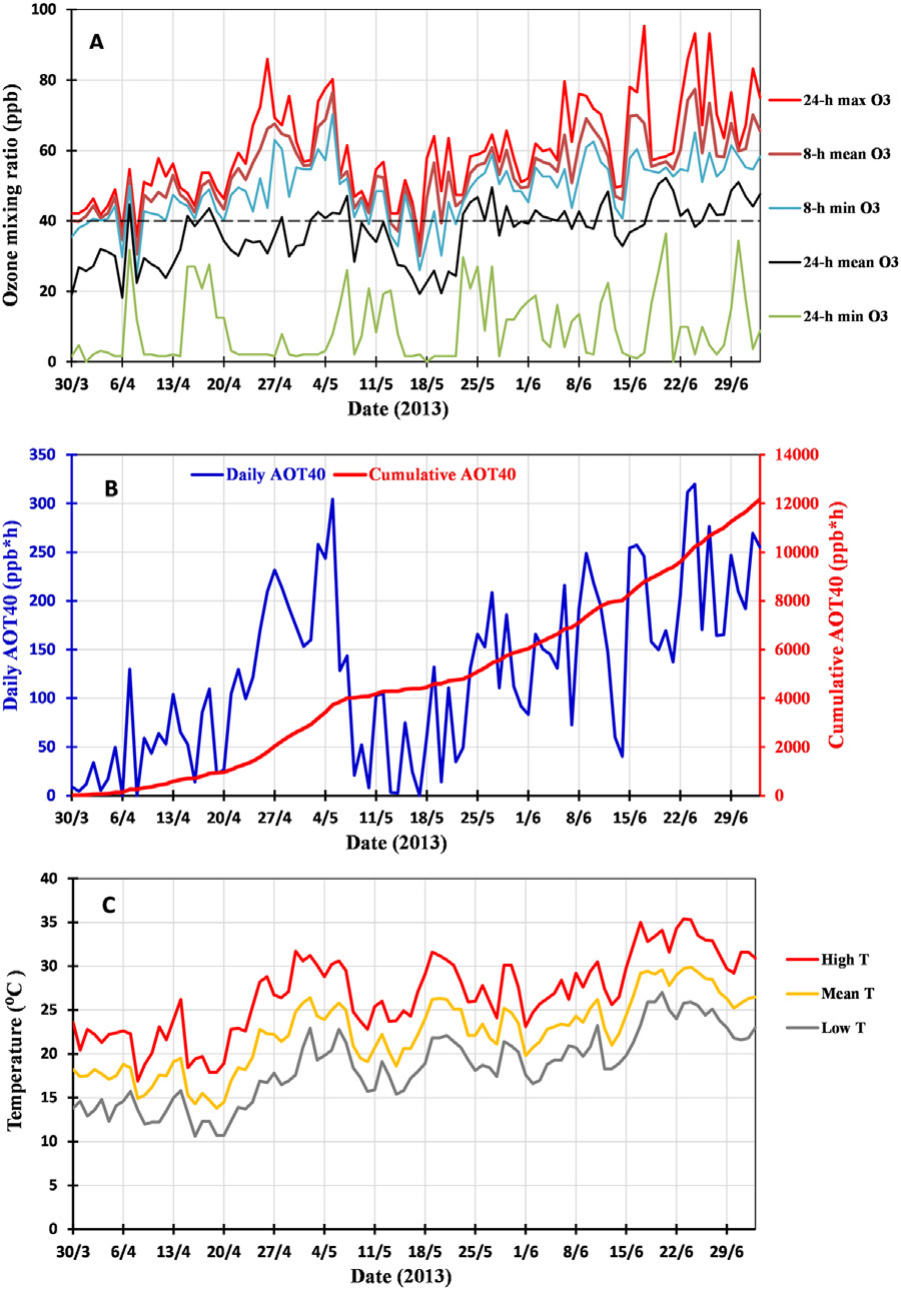
Ozone (O_3_) exposures and ambient air temperature (T) during the field experiment. Different metrics are illustrated in different sets of combinations for presentation purposes to permit easy comparisons according to the point of view one looks at them. The uppermost panel, A, displays the maximum daily concentration (24-h max O_3_), average eight-hour concentration (8 h mean O_3_), lowest among the high concentrations of the eight hours (i.e. the 8th highest value) (8-h min O_3_), average concentration of 24 h (24 h mean O_3_), and minimum concentration of 24 h (24 h min O_3_); the ozone threshold value of 40 ppb is highlighted with a dashed horizontal line. The middle panel, B, presents the daily value of the accumulated ozone exposure above the threshold of 40 ppb (daily AOT40; right axis), and the cumulative value of AOT40 (× 100; right axis). The lowermost panel, C, illustrates the highest (high T), average (mean T), and lowest (low T) ambient air temperature, occurring during the experimentation period.

Hourly data of ozone and daily mean, lowest, and highest temperature were obtained from a meteorological station at Hymettus (122 m above sea level; 37°57′12″N; 23°44′56″E), approximately 6 km from the experimental field (http://www.ymittosmeteo.gr/). From these data, the accumulated ozone exposure above a threshold of 40 ppb (AOT40) was also calculated as a metric of ozone risks to vegetation, which is also adopted by international regulatory authorities [19,27,28]. According to the 2008/50/CE Directive of the European Union, the threshold for the protection of agricultural crops is set at 3000 ppb.h for the three months of cultivation. In this study, the AOT40 from April to July (4 months), when leaves were sampled during anthesis, was about 12,000 ppb.h, a value that is four times higher than the threshold critical level. In fact, high exposures in June alone led to an AOT40 two times higher than the threshold critical level based on three months. The ozone exposures and air temperatures are presented in Fig. 2.

**Fig. 3:**
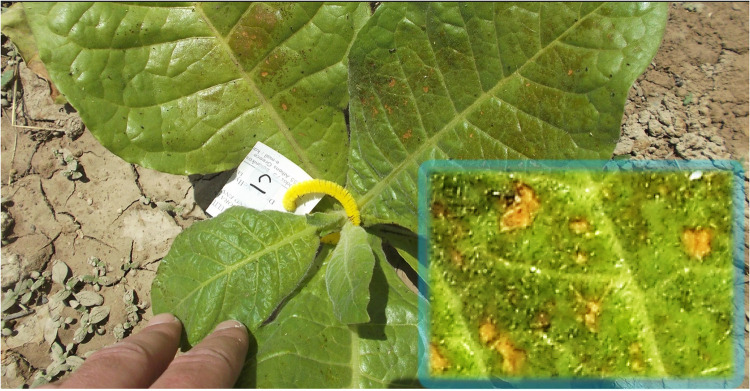
Phytotoxicities induced by the antitranspirant di-1-*p*-menthene (Vapor Gard) on Bel-W3 tobacco plants one day after foliar spray at the concentration of 10%. The inlet presents a magnification of tissue necrosis using +20% brightness and +40% contrast for presentation purposes. Photo courtesy of © Evgenios Agathokleous 2013.

**Fig. 4:**
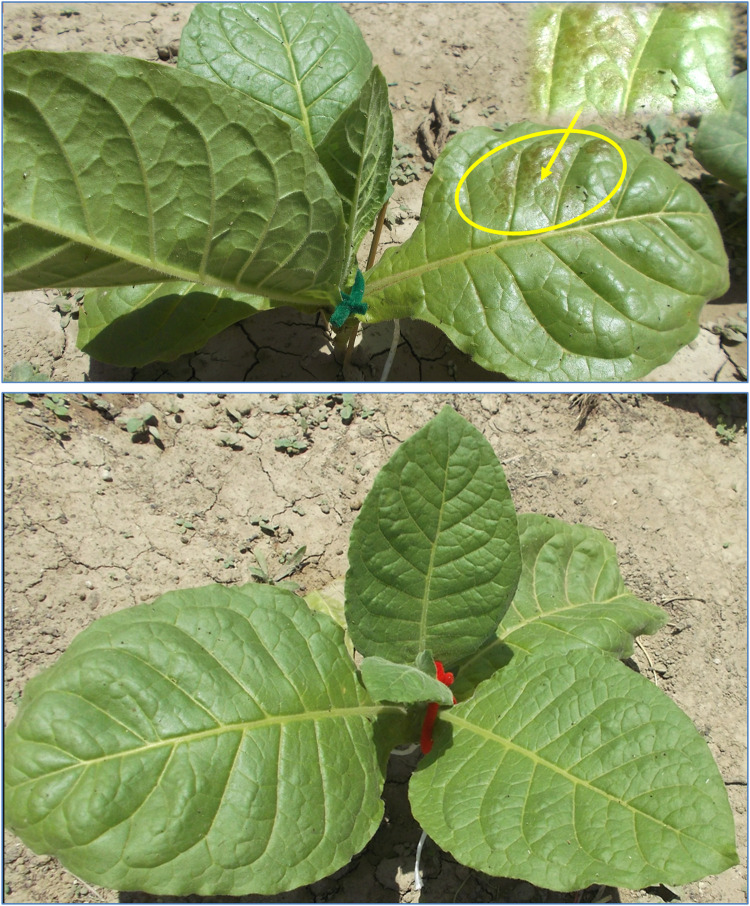
Bel-W3 tobacco plants one day after foliar spray with the antitranspirant di-1-*p*-menthene (Vapor Gard) at the concentrations of 5% (upper photo) and 1% (lower photo). The inlet presents a magnification of tissue necrosis using +20% brightness and +0% contrast for presentation purposes. Photo courtesy of © Evgenios Agathokleous 2013.

Upon transplantation, plants were fertilized with high concentrated N-P-K water soluble fertilizer, MAXIGRO 20–20–20 (Fertifarm Chemical Co, Nicosia, Cyprus) according to the manufacturer's recommendation (5 kg acre^−1^). The exact composition was 20% N (5.7% nitrate, 3.9% ammoniacal, 10.4% urea), 20% P_2_O_5_, and 20% K_2_O. On 15 May, due to outbreak of aphids *Myzus persica* Sulzer (Aphididae, Hemiptera), plants were sprayed with a customized insecticide (5 ml of commercial dishes soap and 30 ml alcohol mixed in 1 L of water). The application was successful since there were no more aphids two days later and the plants did not show any toxicity symptoms. On 21 May, plants were randomly allocated to different chemical treatments and sprayed by hand until run-off. Plants were sprayed with water, the antiozonant ethylenediurea (EDU), or the antitranspirant di-1*-p*-menthene (Vapor Gard), a natural terpene polymer (pinolene). Vapor Gard (Intrachem Bio Italia, Cesena, IT) is consisted of 96% di-1-*p*-menthene active ingredient and 4% inert Ingredients. One day later, toxicity symptoms were apparent on leaves treated 10% Vapor Gard (Fig. 3) and at an apparently smaller extent in those treated with 5% Vapor Gard (Fig. 4; upper). No such apparent symptoms appeared in plants treated with 1% Vapor Gard (Fig. 4; lower), water, and EDU. Vapor Gard toxicities on Bel-W3 plants were also observed in a different chamber experiment with small seedlings treated with 1 or 5% Vapor Gard [29]. Considering that lower concentrations of Vapor Gard did not induce apparent toxicities in this experiment, these results suggest that older/bigger Bel-w3 plants are more tolerant to Vapor Gard phytotoxicity, under ambient conditions, than younger/smaller plants grown in controlled environment experimental chambers. Also, Bel-W3 plants may be more sensitive to Vapor Gard toxicity than the sensitive snap bean bioindicator genotype [29,30]. Although the Vapor Gard-induced leaf injuries can be remarkably different from the ozone-induced foliar visible injuries of Bel-W3 plants [29], these results highlight that incorporation of such chemical treatments in ozone biomonitoring systems should be done with caution and based on proper dose-response evaluations of the chemical solutions and understanding of their individual effects before application within biomonitoring systems. This study did not aim at evaluating the effects of chemical solutions per se, but at providing a variable pool of plant material that can be used for more representative predictive model of leaf area based on a diverse plant pool and considering that such chemical solutions are often applied within biomonitoring systems. However, it is important to clarify these findings to improve the methodology of new studies, potentially save resources and time, and decrease the odds for experimental error introduced by chemical solution side effects. Due to the apparent phytotoxicity of Vapor Gard, the chemical treatments were conducted once without repeating them. Throughout the field experiment, plants were irrigated regularly, as needed to keep the soil moisturized, and weeds were manually removed by hand (30 May and 5 July).

After flowering (61 days after transplanting to the experimental plot), 138 leaves of various sizes (excluding the most basal at progressed senescence) were excised with a scissor from 40 randomly selected plants from all the five chemical solution treatments. The length and the maximum width of each leaf were measured with a ruler with an accuracy of 1 mm. Then, photos were taken from a fixed position, using a digital camera and processed with image analysis software (Adobe Photoshop CS4 Extended v.11, Adobe Systems Incorporated, CA-USA). Photos were first converted into Gray Scale mode, while the measurement scale was set to centimeters (cm) for each photo separately, according to a known fixed distance on a ruler placed near each leaf when photos were taken. Then, we measured the area of each leaf (Adobe Photoshop CS4 Extended v.11, Adobe Systems Incorporated, CA-USA). For each leaf we also calculated the product of width and length (Width * Length). In this method, the length indicates the farthest distance from the lowest point of the leaf blade to the highest point of the leaf blade. Similarly, the width indicates the distance between the two farthest points vertically on the leaf blade, i.e. the widest point of the leaf blade.

## Model development

**Fig. 5:**
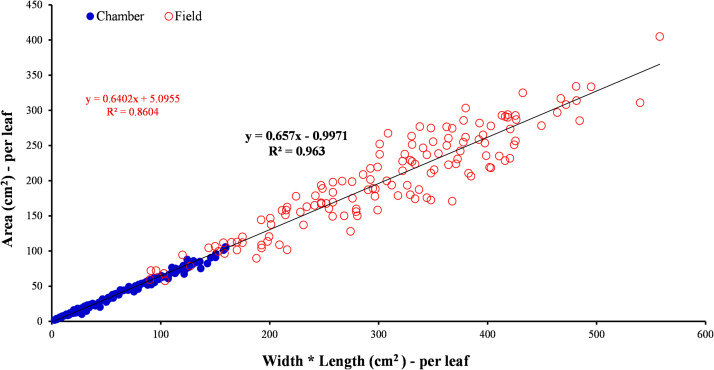
Predictive model of leaf area of Bel-W3 tobacco plants. Individual leaf area is predicted by the product of leaf blade width and leaf blade length. The field data are obtained from big ground-grown plants in the experiment newly reported in this paper whereas the chamber data were obtained in an earlier experiment conducted in charcoal-filtered and ozone-enriched walk-in closed chambers in the laboratory [29]; details are reported in that study.

To develop the simple linear regression of Area and Width*Length of each leaf, the two variables were plotted, and the regression line was added (Fig. 5). Width*Length serves as a predictor variable (independent), thus placed on the x-axis and area serves as the variable criterion (dependent), thus placed on the y-axis. The result was a very strong positive linear regression (*r* = 0.928), with b_1_=0.6402 and se=5.0955 (Fig. 5). The equation for the linear regression is *y*= (β_1_x) +β_0_ where β_0_ represents the estimate of the mean outcome when *x* = 0.and b is the intercept.

**Fig. 6:**
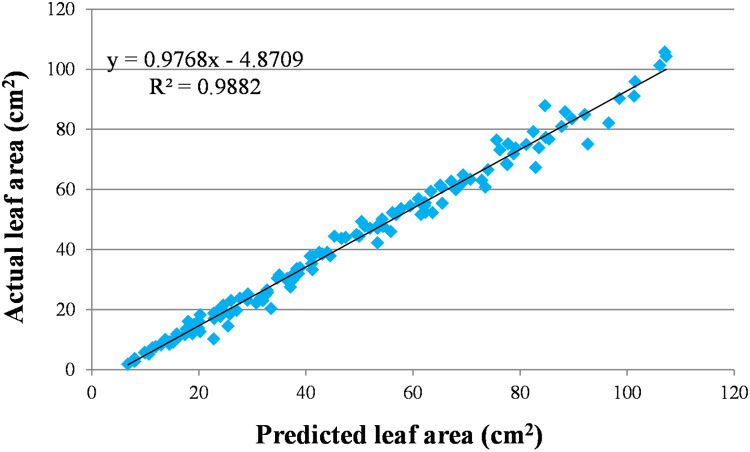
Performance of the predictive model of leaf area of Bel-W3 tobacco plants. The model developed in this study was applied to predict the leaf area of Bel-W3 leaves obtained in an earlier experiment [29] conducted in charcoal-filtered and ozone-enriched walk-in closed chambers in the laboratory (x axis). The predicted values are shown relative to the values of the actual leaf area measured empirically (y axis).

To evaluate whether the generated predictive model is accurate, we applied it to the Width*Length data obtained in a previous experiment [29] with Bel-W3 plants grown in walk-in closed chambers filtered with charcoal or enriched with ozone in the laboratory (Fig. 6). Then, we compared the predicted values with the actual values of leaf area measured in that study [29]. The fitting was good (Fig. 6); however, the model obtained from the experiment in this study overestimated the actual leaf area by a factor of 1.282 ± 0.339 (SD; *n* = 138). This is assumed to be because the data obtained in the earlier experiment come from tiny seedlings grown in 0.5 L pots for a short time (12-day experiment), whereas the data in this study come from ground-grown plants (no root growth limitation) that grew tall and matured.

Due to the aforementioned overestimation, we incorporated the data of the previous study [29] in the predictive model to evaluate whether an improved outcome can be obtained. This resulted to a near perfect linear regression (*r* = 0.981), with b_1_=0.657 and se=−0.9971 (Fig. 5).Then, this model was applied to predict the leaf area of both previous chamber trial and present field experiment. The performance of the model in predicting the actual leaf area, in terms of regression coefficient, was identical to that obtained when the specific model of each respective study was applied. However, this integrated predictive model led to a considerable decrease in bias (the under- or over-estimation) of the values for either experiment. Specifically, compared to the overestimation by a factor of 1.282 on average when only the data from the present experiment were used, application of the model integrating the data from both experiments lead to an overestimation of 1.003 ± 0.137 (SD; *n* = 138) and 1.009 ± 0.138 (SD; *n* = 138) for the previous chamber and present field experiments, respectively. Considering that the 95% CI of both estimates was 0.023, most predicted values had a theoretically negligible deviance from the actual values. Therefore, this integrated predictive model can be used to predict the leaf area of Bel-W3 plants of various sizes, from the smallest to the biggest ones.

Regarding the weakness of the previous model, which was generated using small plants and had limited accuracy in predicting the leaf size of larger plants, it is worth noting that, in general, the prediction of a model is reliable only within the range of values of the independent variable (predictor, X) from which its parameters were generated. Thus, the previous model was useful for predicting the leaf size of small plants, while the new improved model developed in this study incorporates data from both small and large plants, allowing for accurate predictions across the full range of leaf sizes found in tobacco Bell-W3 plants.

## Conclusions

This study leads to some main conclusions:

The new field experiment conducted with ground-grown Bel-W3 plants in the framework of this study documented that pinolene (Vapor Gard) causes phytotoxicities when applied at 5% or 10% but not at 1%. Hence, bigger field-grown plants are less sensitive to pinolene than small chamber-grown plants. Considering that pinolene was tested in ozone-enriched closed chambers in previous studies, the findings of the present study encourage further evaluations of the potential of pinolene smaller than 5% for use within Bel-W3 ozone biomonitoring.

Using data of leaf area obtained from large plants in this field experiment combined with data obtained from small plants in a previous chamber experiment, an integrated predictive model was developed. This model efficiently predicts individual leaf area of all sizes of Bel-W3 leaves, from the tiniest to the largest, using data of the width and length of leaves from non-destructive measurements. The total plant leaf area can be thus easily estimated in ozone biomonitoring projects without destructing the foliage or needing instruments or devices. This method can help to save important time as well as precious plant material for further analyses that may be needed. Beside its scientific and environmental value, this method can be used for environmental and scientific education of the public, and within a citizen science approach, engaging the public in environmental research and its importance as well as to science and its method. Its easiness and simplicity makes it suitable for students at various levels and those attained a basic level of compulsory education. In addition to environmental education and introduction to science and its method, such simple ‘exercises’ can be used for hands-on experience to showcase the importance and applications of mathematics since a common phenomenon in many people is math anxiety and thus poor math skills [31,32].

## Ethics statements

This study did not involve human subjects, animal experiments, or data collected from social media platforms

## CRediT authorship contribution statement

**Evgenios Agathokleous:** Conceptualization, Investigation, Supervision, Writing – original draft. **Kun Zhang:** Data curation, Writing – original draft, Visualization. **Costas J. Saitanis:** Conceptualization, Data curation, Visualization, Writing – review & editing.

## Declaration of Competing Interest

The authors declare that they have no known competing financial interests or personal relationships that could have appeared to influence the work reported in this paper.

## Data Availability

Data used are reported in figures within the article.
